# Correlation Between IVIM-DWI Parameters and Pathological Classification of Idiopathic Orbital Inflammatory Pseudotumors: A Preliminary Study

**DOI:** 10.3389/fonc.2022.809430

**Published:** 2022-03-11

**Authors:** Jian Pu, Yi Liang, Qian He, Ju-Wei Shao, Min-Jie Zhou, Shu-Tian Xiang, Ying-Wen Li, Jian-Bo Li, Shun-Jun Ji

**Affiliations:** ^1^ Radiology Department, Affiliated Hospital of Yunnan University, Kunming, China; ^2^ Radiology Department, Shaanxi Province Tumor Hospital, Xi’an, China; ^3^ Medical Imaging, Kunming Medical University, Kunming, China

**Keywords:** orbital cavity, inflammatory pseudotumor, magnetic resonance imaging, incoherent motion in voxels, pathology, typing

## Abstract

**Objective:**

To investigate the correlation between intravoxel incoherent motion diffusion-weighted imaging (IVIM-DWI) and the pathological classification of idiopathic orbital inflammatory pseudotumors (IOIPs).

**Methods:**

Nineteen patients who were diagnosed with IOIPs (a total of 24 affected eyes) between November 2018 and December 2020 were included in the study. All the patients underwent magnetic resonance imaging orbital plain scans and IVIM-DWI multiparameter scans before an operation. The true diffusion coefficient (D), pseudodiffusion coefficient (D*), and perfusion fraction (f) values were obtained. Based on histopathology, the lesions were divided into three types: lymphocytic infiltration, fibrosclerotic, and mixed. The correlation between IVIM-DWI parameters and pathological classification was tested with the histopathological results as the gold standard. The data were analyzed using SPSS version 17.0, with P < 0.05 defined as significant.

**Results:**

Among the 19 patients (24 eyes) affected by IOIP, there were no significant differences between IOIP pathological classification and gender or age (P > 0.05). There were statistically significant differences between the D and f values for different pathological types of IOIP and IVIM parameters (P < 0.05), and there was no significant difference in D* value between the different pathological types (P > 0.05).

**Conclusion:**

The D and f values showed correlation with different types of IOIP, and the sensitivity of the D value was higher than that of the f value. The D* value showed no significant distinction between pathological types of IOIP.

## Introduction

Idiopathic orbital inflammatory pseudotumors (IOIPs) are a non-specific type of orbital inflammatory disease. Orbital inflammatory disease is the third most prevalent type of orbital lesion (1), accounting for 9% of cases (2). According to the different proportions of chronic inflammatory cell infiltration and fibrous tissue hyperplasia in the pathological tissues, IOIP can be classified into a diffuse lymphocytic infiltration type, a mixed type, and a fibrosclerotic type (3). The pathological type is closely related to the choice of treatment method (4). The current treatment options include corticosteroid therapy, surgical resection, and radiology therapy (5, 6). The lymphocytic infiltration type is sensitive to corticosteroid therapy, while the mixed and fibrosclerotic types show poor response to corticosteroid therapy and have high recurrence rates (7, 8). Radiotherapy has a good effect on IOIPs that are unresponsive to corticosteroid therapy, cannot be surgically treated, and/or show recurrence after surgical resection (5, 9). Therefore, it is particularly important to clarify the pathological classification of IOIPs before clinical intervention (10, 11) to help select the most suitable clinical treatments.

Histopathology, representing the gold standard for the diagnosis and classification of IOIPs (3, 12), requires biopsy or surgical resection of the diseased tissue. These procedures are invasive examination methods (13), and incorrectly performed operations may result in orbital soft tissue injury such as damage to the ophthalmic muscle and optic nerve. Therefore, it is urgent to find an alternative, noninvasive, diagnostic method. Magnetic resonance imaging (MRI) scanning provides high tissue resolution images, is noninvasive, and does not require radiation; for these reasons, it is widely used in the diagnosis and treatment evaluation of orbital diseases. In recent years, MRI scanning has been routinely carried out in the diagnosis of IOIPs in clinical settings (14). The application of contrast-enhanced and various functional imaging techniques in the differential diagnosis of IOIPs has become a key area of research (15–17).

Intravoxel incoherent motion diffusion weighted imaging (IVIM-DWI) is a functional imaging technique of magnetic resonance examination (18). Based on the double exponential model, the following parameters can be obtained: true diffusion (D), false diffusion coefficient (D*), and perfusion fraction (f). D value is pure diffusion coefficient, representing pure water molecular diffusion motion (slow diffusion motion component), also known as slow cell diffusion, in mm^2^/s; D* is the false diffusion coefficient produced by blood circulation, which represents the incoherent movement of microcirculation in the voxel (the diffusion movement related to perfusion, or the rapid diffusion movement), and the unit is mm^2^/s; The f value is the perfusion fraction, which represents the volume ratio of perfusion effect diffusion in microcirculation of voxel to the overall diffusion effect, and the value is between 0-1. Previous studies have mostly used DWI and ADC values to diagnose or distinguish benign and malignant head and neck tumors, DWI cannot distinguish between the diffusion of water molecules and the perfusion of blood (19–22). Specifically, intravoxel incoherent motion diffusion-weighted imaging (IVIM-DWI) can simultaneously quantify the diffusion of water molecules and microcirculation perfusion in living tissues, and thus compensates for the limitations of traditional DWI. Taking readings of these measures can provide a comprehensive picture of the quantitative parameters of water molecule movement in tissue cells and blood microcirculation, and the procedure does not interfere with the diffusion movement of water molecules outside tissue cells (23). IVIM-DWI has high diagnostic and differential diagnostic value for liver, kidney, and pancreatic diseases (24–26), and it is a reliable method for distinguishing orbital lymphoma and IOIPs (1).

In this study, we analyzed the correlation between the pathological classification and the IVIM-DWI parameters of IOIPs and evaluated the diagnostic efficacy of the different parameters in IOIP classification.

## Materials and Methods

### Patients

**Figure 1:**
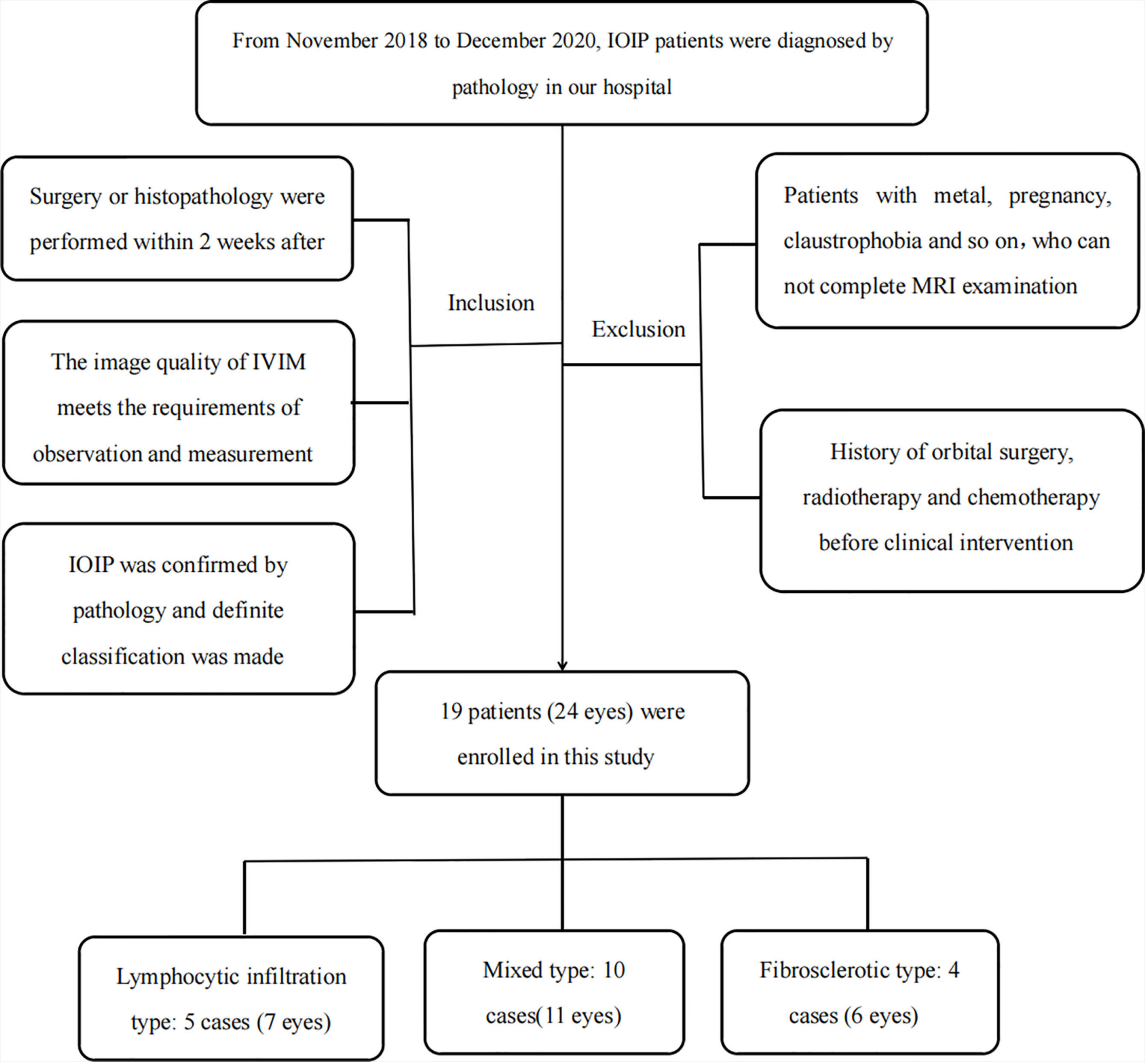
Flowchart showing the patient recruitment process for the current research.

The sample group consisted of patients with IOIPs diagnosed by pathology between November 2018 and December 2020. Routine MRI and IVIM-DWI scans were performed before clinical intervention in all cases, and biopsy or surgical resection procedures were performed within two weeks after the MRI examination. The location of the biopsy is the center of the largest slice of the mass. The patients were divided into three groups according to their IOIP pathological results: lymphocytic infiltration, mixed, and fibrosclerotic types. The exclusion criteria were a patient having undergone surgery, radiotherapy, or chemotherapy before examination, a lack of pathological examination, or no pathological classification. A final total of 19 patients (24 eyes) was included in the study (**Figure 1**).

### Image Data Acquisition and Processing

#### MRI Scanning Parameters

Conventional MRI and IVIM-DWI were performed with a GE signal 1.5T HDX superconducting MR scanner (8-channel head coil). Plain scan sequences included cross-sectional T2WI, T1WI, fat suppression T2WI, coronal T2WI, single-index DWI, and double-index IVIM. The flat scan matrix was 224 × 160, repetition time (TR) was 3,000 ms, echo time (TE) was 90 ms, field of view (FOV) was 24 × 24 cm, number of excitations (NEX) was l, layer thickness was 4 mm, layer spacing was 1 mm, the number of layers was 14, and the turning angle was 15°. Single-shot spin echo planar imaging was used for DWI scanning, with b value of 1,000 s/mm^2^ (TR: 3,500 ms, TE: 75 ms), matrix of 256 × 256, and FOV of 18 × 18 cm. For axial IVIM, the parameters were as follows: b values 0, 20, 50, 75, 100, 150, 200, 400, 800, 1,000, 1,200, 1,500, and 2,000 s/mm^2^, TR 5,098 ms, TE 69 ms, layer thickness 4 mm, interval 1 mm, matrix 140 × 125, FOV 24 × 24 cm, NEX 1, and number of layers = 20.

#### Postprocessing of MRI Image

**Figure 2:**
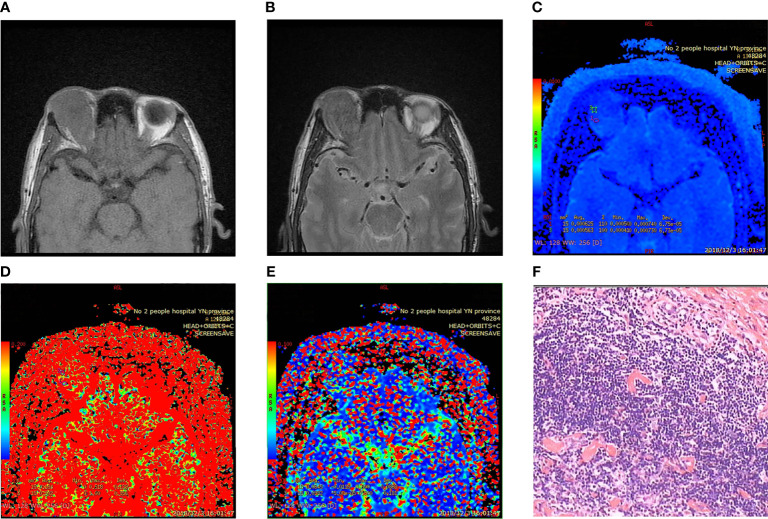
Fibrosclerotic idiopathic orbital inflammatory pseudotumor. **(A, B)** MRI showing isointensity on T1WI and slight hypointensity on T2WI. **(C–E)** Pseudo color IVIM image: D value = 0.594 × 10^−3^ mm^2^/s; D * value = 81.4 × 10^−3^ mm^2^/s; f value = 24.3%. **(F)** Microscopic collagen fibers proliferated significantly, and lymphocytes infiltrated between fibers and muscle bundles (HE × 400). Immunohistochemistry: CD3 (+) CD20 (+) CD38 (+) CD68 (+) Pax-5 (+) mum-1 (+) Bcl-2 (+) bcl-6 germinal center (+) CD79a (+) Ki-67 (+) 30% CD138 (+).

The MR multisequence plain scan and IVIM sequence scan data were collected. The acquired images were analyzed and postprocessed by two imaging experts on ADW workstation READY view software. The region of interest (ROI) was defined as the most uniform area of the abnormal signal area of the lesion, selected manually to determine the scope of the lesion and to avoid the cystic area and necrotic area. The ROI area was required to be smaller than the whole lesion, and an average value was taken from three measurements. The ROI selected in each case therefore varied according to the size of the measured object, with areas ranging from 8 to 10 mm^2^. The ROI selected by the image measurement is the pathological sample region. The parameters D, D*, and f were measured using inclined view software (**Figure 2**).

### Statistical Methods

SPSS version 17.0 was used to analyze the data. Statistics for the age of participants were calculated in the form mean ± standard deviation, and sex using the Pearson chi-square test. Pearson correlation analysis was applied to analyze the consistency of the measurement data reported by the two experts, and the correlation coefficient was >0.4. The correlations between pathological classification and the IVIM parameters D, D*, and f were analyzed by ANOVA (P < 0.05). The least significant difference method and receiver operating characteristic (ROC) curve analysis were used to calculate pairwise comparisons between groups.

## Results

### Patients’ Clinical Data

Among the 19 patients in the sample, there were 10 males and 9 females. All the patients were of Han nationality, aged between 23 and 75, with an average age of 52.32 ± 15.71 years. There were 5 patients (7 eyes) with the lymphocytic infiltration type (4 females and 1 male, age range 44–70, average age 58.2 ± 11.71 years), 10 patients (11 eyes) with the mixed type (3 females and 7 males, age range 23–75, average age 54.8 ± 15.02 years), and 4 patients (6 eyes) with the fibrosclerotic type (2 females and 2 males, age range 26–64, average age 38.75 ± 17.23 years). There was no correlation between sex, age, and pathological type of IOIP (P > 0.05) (**Table 1**).

**Table 1:** Clinical data and pathological classification of 19 patients (24 eyes) with IOIP.

General information	Pathological classification			F value	P value
	Lymphocytic infiltration type (n = 5)	Mixed type (n = 10)	Fibrosclerotic type (n = 4)		
Age	58.20±11.71	54.80±15.02	38.75±17.23	0.607	0.779
Gender					0.187
Male	1	7	2		
Female	4	3	2		

### Correlation of D, D*, and f Values With Pathological Classification of IOIP

The D values of the IVIM parameter were 0.475 ± 0.030 × 10^−3^ mm^2^/s in the lymphocytic infiltration type, 0.506 ± 0.049 × 10^−3^ mm^2^/s in the mixed type, and 0.628 ± 0.033 × 10^−3^ mm^2^/s in the fibrosclerotic type. The D* values were 59.437 ± 22.576 × 10^−3^ mm2/s in the lymphocytic infiltrating type, 52.192 ± 32.191 × 10^−3^ mm^2^/s in the mixed type, and 31.410 ± 28.868 × 10^−3^ mm^2^/s in the fibrosclerotic type. The f values were 24.490 ± 54.522 in the lymphocytic infiltration type, 33.466 ± 75.004 in the mixed type, and 39.655 ± 79.637 in the fibrosclerotic type. The lowest D and f values were observed in the lymphocytic infiltration type and the highest in the fibrosclerotic type, with the mixed type falling between the other two groups. There were significant differences between the different pathological types (P =0.000). The analysis of variance of D value and f value in IOIP typing showed that the F values were 25.464 and 7.605, respectively. The highest D* value was observed in the lymphocytic infiltration type and the lowest in the fibrosclerotic type. There were no significant differences between D* values for the different IOIP pathological classifications (P =0.126) (**Table 2**).

**Table 2:** Correlation between different pathological types and IVIM parameters in 19 female patients (24 eyes) with IOIP.

IVIM parameters	IOIP pathological classification			F value	P value
	Lymphocytic infiltration type	Mixed type	Fibrosclerotic type		
D (×10-3mm2/s)	0.475±0.030	0.506±0.049	0.628±0.033	25.464	0.000
D* (×10-3mm2/s)	59.437±22.576	52.192±32.191	31.410±28.868	1.630	0.126
f (%)	24.490±54.522	33.466±75.004	39.655±79.637	7.605	0.007

D, true diffusion; D*, false diffusion coefficient; f, perfusion fraction.

### Diagnostic Efficacy of D Value and f Value in Different Types of IOIP

**Figure 3:**
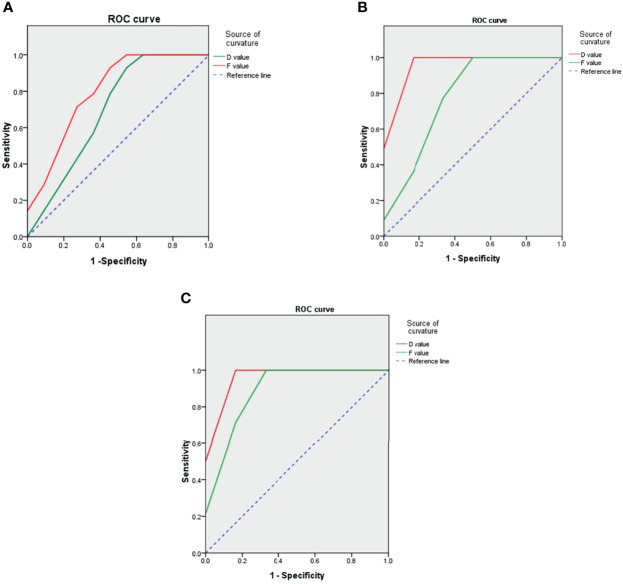
ROC curve showing that both D value and f value can effectively distinguish between lymphocytic infiltration and fibrosclerosis types **(C)**, D value cannot effectively distinguish between lymphocytic infiltration and mixed types **(A)**, and f value cannot effectively distinguish between mixed and fibrosclerosis types **(B)**. **(A)** Lymphocytic infiltration type and mixed type. **(B)** Lymphocytic infiltration type and fibrosclerotic type. **(C)** Lymphocytic infiltration type and fibrosclerotic type.

The ROC curve (**Figure 3**) combined with Youden index analysis results (**Table 3**) shows that the threshold values of D were 0.550 × 10^−3^ mm^2^/s and 0.574 × 10^−3^ mm^2^/s. The sensitivity and specificity of differentiation between the lymphocytic infiltration and fibrosclerotic types and between the mixed and fibrosclerotic types were 100%. The D value could not effectively distinguish the lymphocytic infiltration type from the mixed type (P = 0.174). The threshold of the f value was 32.3 × 10^−3^ mm2/s, and the sensitivity and specificity were 45.5% and 54.5%, respectively. The threshold value was 34.35 × 10^−3^ mm^2^/s, and the sensitivity and specificity were 100% and 83.3%, respectively. The f value could not effectively distinguish between the mixed type and the fibrosclerotic type (P = 0.056).

**Table 3:** Efficacy of IVIM parameters in differentiating IOIP pathological types.

	D value					f value				
	threshold (×10-3mm 2/s)	P value	Susceptibility(%)	Specificity(%)	Threshold (×10-3mm 2/s)	AUC value	P value	Susceptibility(%)	Specificity(%)
Lymphocytic infiltration type and mixed type			0.174			32.3	0.805	0.033	45.5	54.5
Lymphocytic infiltration type and fibrosclerotic type	0.550	1	0.003	100	100	34.35	0.905	0.015	100	83.3
Mixed type and fiber hardening type	0.574	1	0.001	100	100			0.056		

D, true diffusion; f, perfusion fraction.

AUC, Area Under ROC Curve.

## Discussion

Although IOIPs are considered a benign condition, severe cases where the disease progresses can eventually result in substantial orbital fibrous connective tissue hyperplasia, orbital casting, and loss of vision. Therefore, early diagnosis and pathological classification and the implementation of individualized treatment are particularly important (10). The histopathological features of IOIP are infiltration of chronic inflammatory cells (including lymphocytes, plasma cells, and eosinophils) accompanied by different degrees of fibrous connective tissue hyperplasia. The pathological type is based on the proportions of the different tissue components. The pathological change in the lymphocytic infiltration type is primarily due to lymphocyte proliferation. Proliferative lymphocytes are closely arranged, and the tissue structure is relatively uniform and mixed with a small amount of fiber components. The main pathological changes in the fibrosclerotic type involve the proliferation of collagen fibers with a small number of cellular components. Mixed-type pathological changes involve mixed collagen fibers and chronic inflammatory cells, and it is determined by the mix of these two factors (27).

In this study, the D and f values were used to identify IOIP pathological classifications. The D value is the true diffusion coefficient, which is dependent on the limited diffusion of intracellular and extracellular water molecules caused by the cell membrane, macromolecules, and fibrosis (28). The f value is the perfusion fraction, which reflects the distribution characteristics of local blood vessels in the tissue and is positively correlated with the number of capillaries in the tissue (29). The elements of the pathological basis of the different types of IOIP are the different proportions of tissue components, the restricted diffusion of water molecules in the tissues, and the distribution characteristics of the local blood vessels; therefore, the D and F values may help to distinguish between the different types of IOIP. In our study, there were no statistically significant between the D* values for the different types. The D* value is the perfusion-related diffusion coefficient, which is a pseudodiffusion coefficient that indicates the diffusion effect caused by microcirculation perfusion in the local ROI and is affected by the number and size range of the selected b value. In a study of the liver, Riexinger et al. (24) obtained different D* values by using different b values. Chabert et al. (30) showed that there was no significant difference in the coefficient of variation of the D value in healthy subjects’ brain IVIM parameters, while the coefficient of variation of the D* value decreased by approximately 39% in healthy subjects’ brain IVIM parameters. In the current study, no difference was observed between the D* values for the different IOIP types, which may be related to the degree of variation in the D* value. Federau et al. (31) found that the D* value obtained in systole was greater than that in diastole. In this study, we found that D * value was not related to IOIP typing, and the conclusion is consistent with it. Furthermore, since ECG gating was not used in our IVIM scanning, the effect of the cardiac cycle on the D* value could not be excluded.

In the current study, the lowest D and f values were observed in the lymphocytic infiltration type and the highest in the fibrosclerotic type, with those for the mixed type falling in between the other two values. We speculate that the degree of restriction of water molecule diffusion by lymphocytes was more significant than by collagen fibers and that the microcirculation perfusion of capillaries between collagen fibers was slightly more abundant than between lymphocytes.

We used ROC curves to compare the diagnostic efficiency of IVIM with different parameter values in IOIP classification. The results showed that the D and f values could effectively distinguish between the lymphocytic infiltration and fibrosclerotic types. This was due to the large difference in the proportion of tissue components between the two types. Lymphocytic tissue showed a low signal on T1WI and a slightly high signal on T2WI, while fibrous tissue showed a low signal on T1WI and T2WI (32), and so it was easy to distinguish between the two. The proportions of tissue components in the mixed type fell between those seen in the other two types, and there was little difference between the mixed type and the lymphocytic infiltrating type or the fibrosclerotic type. Our results also show that the D value could not distinguish between the lymphocytic infiltration type and mixed type while the f value could; in contrast, the D value could distinguish between the mixed type and fibrosclerotic type while the F value could not. Therefore, we speculate that the lymphocytic infiltrating type and the mixed type are mainly composed of lymphocytes, with little difference in the limited degree of water molecules inside and outside the cells, which can be distinguished by the f value as it reflects blood flow microperfusion. The fibrosclerotic and mixed types are mainly composed of collagen fibers, with little difference in blood perfusion. They can be characterized instead by a D value, which reflects the diffusion of water molecules inside and outside the cell with high sensitivity and specificity.

## Study Limitations

There are some limitations to this study that should be mentioned. First, the sample size was limited. These are preliminary results, and the number of cases will continue to expand in the future. Second, ECG gating was not used in this study; therefore, whether it was the effect of the cardiac cycle that led to there being no differences between the D* values for the different IOIP types needs further study.

## Conclusion

Significant differences were observed between the parameters of IVIM-DWI and the parameters of the lymphocytic infiltration, mixed, and fibrosclerotic types of IOIP. The D value and f value are effective in differentiating between the pathological types of IOIP.

## Data Availability Statement

The original contributions presented in the study are included in the article/supplementary material. Further inquiries can be directed to the corresponding author.

## Ethics Statement

The studies involving human participants were reviewed and approved by Ethics Committee of Affiliated Hospital of Yunnan University. The patients/participants provided their written informed consent to participate in this study.

## Author Contributions

Conception and design of the research: QH and JP. Acquisition of data: M-JZ, J-BL, and S-JJ. Analysis and interpretation of the data: JP, YL, and J-WS. Statistical analysis: JP and J-WS. Obtaining financing: S-TX. Writing of the manuscript: JP. Critical revision of the manuscript for intellectual content: QH, Y-WL, and S-TX. All authors contributed to the article and approved the submitted version.

## Conflict of Interest

The authors declare that the research was conducted in the absence of any commercial or financial relationships that could be construed as a potential conflict of interest.

## Publisher’s Note

All claims expressed in this article are solely those of the authors and do not necessarily represent those of their affiliated organizations, or those of the publisher, the editors and the reviewers. Any product that may be evaluated in this article, or claim that may be made by its manufacturer, is not guaranteed or endorsed by the publisher.
